# Validation of evidence-based questionnaire for TCM syndrome differentiation of heart failure and evaluation of expert consensus

**DOI:** 10.1186/s13020-023-00757-1

**Published:** 2023-06-09

**Authors:** Alice Yeuk Lan Leung, Jialing Zhang, Chun Yin Chan, Xiaotong Chen, Jingyuan Mao, Zhenhua Jia, Xinli Li, Jiangang Shen

**Affiliations:** 1 School of Chinese Medicine, University of Hong Kong, 3 Sassoon Road, Pokfulam, Hong Kong, Hong SAR People’s Republic of China; 2 Department of Cardiovascular Diseases, First Teaching Hospital of Tianjin University of Traditional Chinese Medicine, National Clinical Research Center for Chinese Medicine Acupuncture and Moxibustion, Tianjin, 300381 China; 3 National Key Laboratory of Collateral Disease Research and Innovative Chinese Medicine, Shijiazhuang, China; 4 Hebei Yiling Hospital, Key Disciplines of State Administration of TCM for Collateral Disease, Shijiazhuang, China; 5 Department of Cardiology, The First Affiliated Hospital with Nanjing Medical University, Guangzhou Road 300, Nanjing, 210029 China

**Keywords:** Syndrome differentiation, TCM syndromes, Syndrome element, Heart failure, Evidence-based questionnaire, Tongue analysis, Expert consensus, Traditional Chinese Medicine

## Abstract

**Background:**

Traditional Chinese Medicine (TCM) is widely used to treat heart failure (HF). Syndrome differentiation is a unique and crucial component in TCM practice for guiding disease diagnosis and treatment strategies as well as clinical research. The major bottlenecks in TCM syndrome differentiation are the diversity of the syndrome differentiation criteria and the broad spectrum of syndrome patterns, hindering evidence-based studies for clinical research. In the present study, we aim to develop an evidence-based questionnaire for the diagnosis of HF and establish a definitive set of criteria for syndrome differentiation.

**Methods:**

We designed a TCM syndrome differentiation questionnaire for heart failure (SDQHF) based on the "TCM expert consensus for diagnosis and treatment of heart failure" (expert consensus), literature review, and various clinical guidelines. To test the reliability and efficiency of the questionnaire, we performed a large-scale multiple-center clinical trial with the recruitment of 661 HF patients. Cronbach's alpha was used to assess the internal consistency of the SDQHF. Content validity was conducted through expert review. Principal component analysis (PCA) was applied to evaluate the construct validity. We constructed a proposed model for syndrome differentiation for HF based on the PCA results. Tongue analysis was performed to verify the accuracy of syndromes derived from the proposed model and the expert consensus. An evidence-based practical questionnaire for TCM syndrome differentiation patients was developed and validated with the data from 661 HF patients.

**Results:**

The syndrome differentiation criteria were constructed with five syndrome elements (qi-deficiency, yang-deficiency, yin-deficiency, blood stasis, and phlegm retention). The results revealed good convergent and discriminant validity, satisfactory internal consistency, and feasibility. The significant discoveries include: (1) A total of 91% of the derived TCM syndromes from the proposed model matched with the characterized tongue images of the syndrome patterns; (2) Qi Deficiency Syndrome is the dominant syndrome pattern for HF patients, followed by Yang-Qi Deficiency Syndrome and Qi-yin deficiency Syndrome, and finally, Yin-Yang Dual Deficiency Syndrome; (3) The majority of the HF patients had the combination of Blood Stasis and Phlegm Retention Syndromes; (4) The "Yin-Yang Dual Deficiency" Syndrome was a valid syndrome for HF, suggesting that this syndrome pattern should be included in the criteria for syndrome differentiation; and (5) Through the validation of the expert consensus, several recommendations were proposed to improve the accuracy of syndrome differentiation of HF.

**Conclusions:**

The proposed SDQHF and the criteria could be a reliable and valid tool for syndrome differentiation of heart failure with high accuracy. It is recommended to use the proposed model for evidence-based study on Chinese Medicine to diagnose and treat HF.

*Trial registration number:* The trial was registered at the Chinese Clinical Trial Registry, http://www.chictr.org.cn. (Registration No.: ChiCTR1900021929); Date: 2019-03-16.

**Supplementary Information:**

The online version contains supplementary material available at 10.1186/s13020-023-00757-1.

## Introduction

Heart Failure (HF) is the most common cardiovascular disease and the leading cause of death globally. There are approximately 64.3 million heart failure patients worldwide [1]. HF is an end-stage cardiovascular condition signified by dyspnea and fatigue due to global or regional left ventricular dysfunction, companied with fluid retention, pulmonary and splanchnic congestion, and peripheral edema [2]. About 19.2% of patients were hospitalized or died due to heart failure within a year [3].

Traditional Chinese Medicine (TCM) has a long history of dealing with clinical situations similar to HF. Some TCM formulae, such as Qiliqiangxin, Shencaotongmai, Nuanxin, etc., appear to be effective adjunct therapies with conventional medicine for HF treatment [4]. For example, treatment of Qiliqiangxin (QLQX) Capsule, a patent TCM formula, significantly reduced the serum level of NT-proB-type natriuretic peptide (NT-proBNP), improved cardiovascular functional classification, left ventricular ejection fraction (LVEF), increased 6-min walking distance, improved life quality, and reduced composite cardiac events in HF patients [5]. However, current clinical trials seldom evaluate whether the TCM formulae fit the TCM syndrome patterns. Practically, TCM practitioners would apply different TCM formulae to deal with various syndrome patterns presented in the HF patients at different stages or different individuals. Thus, standardization of TCM syndrome patterns with evidence-based support is crucial for the application of TCM formula in HF treatment.

In TCM, syndrome differentiation is a crucial and comprehensive analysis for diagnosing the clinical information obtained from four primary diagnostic methods (inspection, listening and smelling, inquiry, and palpation), practically guiding the therapeutic strategies. The TCM syndrome pattern, composed of different syndrome elements, can be changed with the clinical manifestations and patient's body conditions during disease progression. The dynamic change of syndrome patterns carries significant research values for evaluating disease progression and therapeutic efficacy. TCM practitioners diagnose syndrome patterns based on syndrome elements, phenotypic features, tongue diagnosis, pulse diagnosis, etc. However, the inconsistent criteria for syndrome differentiation and syndrome definition are the major bottlenecks for the standardization of TCM syndrome diagnosis [6–8], thus hindering the use of TCM formulae for HF treatment. Recently, several consensuses and guidelines were proposed to guide the standardization of syndrome diagnosis for HF in TCM practice [9–11]. It is desirable to validate the effectiveness of these consensuses with evidence-based medicine approaches.

In the present study, to facilitate the TCM syndrome differentiation, we developed a questionnaire for collecting clinical manifestations of HF, which is based on "Consensus from TCM experts on diagnosis and treatment of heart failure”(expert consensus) [12]. We performed a multi-center and randomized clinical trial to test the modified questionnaire, and concomitantly, we verified the effectiveness of the expert consensus. Through this study, we provided an evidence-based and definitive criterion for syndrome differentiation for HF patients.

## Methods/design

### Study design and participants selection criteria

This is a pilot study in conjunction with a clinical trial named QUEST to investigate the TCM syndrome pattern distributions and explore the impacts of syndrome pattern types on the efficacy of a TCM formula for HF patients [13]. The clinical trial involved 131 centers in China and Hong Kong SAR with a target number of patient recruitment up to 3,080, which was calculated according to the PARADIGM-HF study based on the cardiovascular death or hospitalization rate for heart failure [14]. All recruited patients must fulfill the inclusive and exclusive criteria (Appendix: Table 5) and be "clinically stable" by receiving at least two weeks of standardized medication treatment for heart failure according to the local treatment guideline with standard drug type and dosage. These patients must be at least 18 years old with a documented diagnosis of heart failure for at least three months, according to the "China Heart Failure Diagnosis and Treatment Guideline" issued by the Chinese Medical Association Cardiovascular Branch, and met the diagnostic criteria, including LVEF, serum NT-proBNP, NYHA cardiac functional grading, etc. Routine medical examinations were performed, including physical examination, blood/urine routine test, clinical biochemical test, 12-lead electrocardiogram (ECG), etc. All patients must complete a specially designed TCM Syndrome Differentiation Questionnaire for heart failure (SDQHF) (Table 1). The study conducted a face-to-face interview to review the questionnaire with the patients to ensure high response rates. The entire interview lasted for approximately 5–10 min. The data collected was manually recorded in the case report form (CRF) and then input into the Epidata software [15]. As part of the SDQHF, the tongue images of the patients were acquired to facilitate the syndrome differentiation. Given the potential inconsistency of interpretation of pulse diagnosis among different TCM practitioners, we excluded pulse diagnosis for the test of syndrome differentiation.

**Table 1:** Inclusive symptom and body signs for questionnaire validation of heart failure patients

36 items in the SDQHF			
1. Palpitations	10. Coughing with Phlegm	19. Cold Sweating	28. Constipation
2. Chest Tightness	11. Color of phlegm	20. Fear of cold	29. Diarrhea
3. Precordial pain	12. Texture of phlegm	21. Coldness feeling in upper/lower limbs	30. Frequent urination
4. Dizziness	13. Distension feeling in chest/abdomen	22. Feeling the heat in palm or midfoot	31. Amount of urination
5. Shortness of breath	14. Hypochondriac pain or feeling of distension	23. Poor Appetite	32. Distension of jugular vein
6. Gasp for breath	15. Edema	24. Sense of thirstiness	33. Cyanosis of face and lips
7. Posture	16. Tiredness	25. Loss of sleep/insomnia	34. Color of face/complexion
8. Coughing	17. Excessive/spontaneous sweating during daytime	26. Dreaming	35. Color of lips
9. Phlegm in the throat with rutting sound	18. Night sweating	27. Somnolence	36. Location of edema

Item 1–30 were answered by the patients. Item 31–36 were answered based on the observation to the patient by the investigators

### Sample size calculation and sample selection for questionnaire validation

Total 661 HF patients were recruited to evaluate the questionnaire's feasibility, reliability, and validity. The minimum sample size required for this testing is 360, calculated based on the population size of 3,080, a confidence level of 95%, and a 5% margin of error. We also followed the rule of thumb of enlisting a sample size of at least ten times the number of items being analyzed in the questionnaire [16].

A mixed-model consecutive and judgment sampling methodology was adopted for this study [17]. A complete data extract from the electronic data capture (EDC) database was performed on a specific date within the assessment period, which was unknown to all hospitals involved. The data were sorted with criteria based on the hospitals with the recruited numbers of patients in descending order. Samples were selected from the top-listed hospitals with the highest number of patients until the desired sample size was reached. These selected 661 samples were collected from 25 hospitals, covering the streams of western medicine, integrated medicine, and traditional Chinese medicine from different regions of China. Adopting this sampling methodology could ensure the reliability and generalizability of samples as hospitals with more recruitment tend to have more resources and motives for the study, along with better administration to yield higher data quality for better representation [18, 19]. The selection of the recruited hospitals minimized the variations of data collection in different hospitals.

### Demographic and clinical characteristics

Total 661 HF patients were recruited from 25 hospitals in Mainland China between May 16, 2019 and Jun 30, 2020. The baseline characteristics of the sample group are shown in Table 2. The mean age was 63 years, and 71.2% were male. The average course of heart failure was 26 months. The HF etiology included cardiomyopathy (58.6%), coronary artery disease (53%), and hypertensive cardiomyopathy (4%). Most HF patients showed an abnormal electrocardiogram (ECG) (95.5%). The mean plasma NT-proBNP level was at 1626 pg/ml, and the mean left ventricular ejection fraction (LVEF) was at 31%.

**Table 2:** Baseline demographic and clinical characteristics of heart failure patients

Baseline characteristics of HF patients	All (n = 661)
Heart failure duration, months	26.0 (8.0–60.5)
Demographics	
Age, yrs	63.0 (54.0–70.0)
Female	193 (28.8)
Male	468 (71.2)
Age range, yrs	
< 50	107 (16.3)
50–70	461 (70.3)
> 75	88 (13.4)
Race	
Han	610 (92.8)
Others	51 (7.2)
Measurements	
BMI, kg/m^2^	23.9 ± 4.1
Systolic BP, mm Hg	120.0 (107.0–130.0)
Diastolic BP, mm Hg	75.0 (68.0–84.0)
Heart rate, beats/min	77.0 (69.0–87.0)
Active smoker	102 (15.5)
Active alcohol drinker	53 (8.1)
Current heart disease	
Cardiomyopathy	378 (58.8)
Coronary artery disease	342 (53.0)
Hypertensive heart disease	36 (4.0)
Echocardiography measurements	
Abnormal ECG	615 (95.5)
LVEF, %	31.0 (27.0–36.3)
LVEF range, %	
40–49	46 (7.8)
30–39	319 (53.8)
< 30	228 (38.4)
NYHA classification	
II	371 (56.5)
III	263 (40.0)
IV	23 (3.5)
Plasma NT-proBNP, pg/ml	1626.5 (849.3–3573.8)
Plasma NT-proBNP range, pg/ml	
< 1000	206 (31.6)
1001–3000	240 (36.8)
3001–9000	162 (24.8)
> 9000	44 (6.7)

Data are presented as mean ± SD (Standard Deviation), medians and ranges, or numbers (%). *BMI* Body Mass Index, *BP* Blood Pressure, *NT-proBNP* N-terminal pro-brain natriuretic peptide, *LVEF* Left ventricular ejection fraction; *NYHA FC* New York Heart Association functional classification, *EGC* Electrocardiography. Heart Rate refers to the resting heart rate

### SDQHF design

**Fig. 1:**
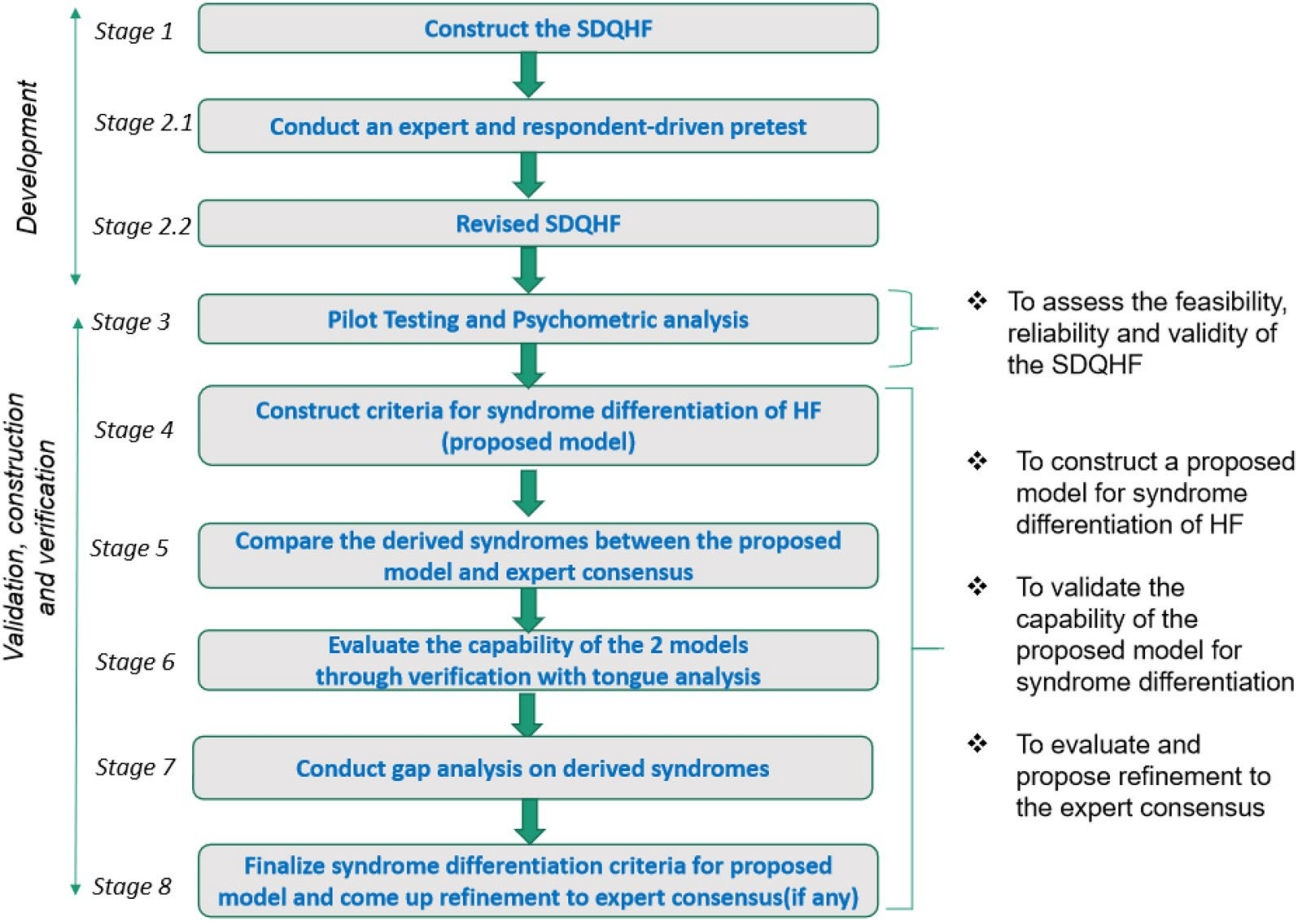
Flow diagram of construction and validation of SDQHF and criteria for syndrome differentiation in heart failure patients

We developed a questionnaire to collect clinical manifestations for HF's diagnosis and syndrome differentiation. Figure 1 shows the workflow of questionnaire development and validation.

#### Constructing SDQHF

The questionnaire was designed according to the criteria of the consensuses, guidelines, and textbooks for diagnosing HF in both TCM and western medicine and integrated with focus group discussion, expert review, and validation [20]. According to TCM Theory, the pathological basis of heart failure could be attributed to the Deficiency of Heart-Qi and Heart-Yang, affecting blood circulation and leading to blood stagnation and body fluid retention, named the retention of phlegm-fluid. Meanwhile, the diuretic treatment and the chronic progression of heart failure could consume Yin-fluids to induce the state of Yin Deficiency. Conclusively, the fundamental cause of heart failure could be related to the Deficiency of Qi and Yang, causing the imbalance and dysfunction of Qi-Blood-Bodily Fluids. Thus, Yin, Yang, Qi, Blood, and Body Fluid are five basic syndrome elements that contribute to the development of heart failure [21]. Accordingly, we designed a TCM Syndrome Diagnosis Questionnaire for Heart Failure (SDQHF) comprising 36 closed-ended items based on various guidelines for heart failure and expert consultation. These guidelines included the "Guideline for TCM diagnosis and treatment of heart failure," "TCM expert consensus for diagnosis and treatment of heart failure" (expert consensus), and the “Guiding principles for a clinical study on new drugs in Traditional Chinese Medicine” [9, 12, 22]. We carefully considered medical terminologies to avoid jargon. We identified the syndrome elements according to fundamental units of TCM syndromes and constructed the syndrome definition of heart failure. We carefully determined the inclusive items for the questionnaire based on the typical clinical symptoms and manifestations from the diagnostic criteria of Cardiology and the sufficient attributes of TCM syndrome differentiation. These 36 items covered primary and concomitant signs and symptoms of heart failure and symptoms requisite for diagnosis and syndrome differentiation of HF. Each item was given a ranked scale with simple descriptions to indicate the severity or frequency of the clinical manifestations, which was constructed under the complexion of items for accurate assessment. In the interview, the patients must answer the first 31 items in the SDQHF about clinical manifestations. The interviewers determined the last five items about the physical signs based on their observations of the patients. The list of the 36 items in SDQHF is shown in Table 1. Tongue images of the patients were acquired to facilitate the syndrome differentiation.

#### Conducting an expert and respondent-driven pretest

A pretest on the TCM questionnaire was conducted by three subject matter experts, including a general physician, a cardiologist, and a TCM practitioner, on a small sample of HF patients with various profiles in age group, social class, and education level. The study aimed to get initial feedback on whether each questionnaire item would genuinely reflect the construct for disease diagnosis and syndrome differentiation of heart failure and to observe any potential issues throughout the interview process.

#### Perform pilot testing for questionnaire validation (psychometric analysis)

Pilot testing was conducted to test the feasibility, reliability, and validity of the SDQHF based on the clinical data obtained from 661 patients. Cronbach's alpha was assessed for internal consistency representing reliability. Construct validity was assessed via convergent and discriminant validity. Content validity was conducted through expert review by TCM practitioners, general physicians, and cardiologists. The psychometric properties and the structural validity of the questionnaire were analyzed by principal component analysis (PCA).

#### Constructing a proposed model for syndrome differentiation of HF

The PCA analysis facilitated the streamlining of the questionnaire to be more compendious in syndrome differentiation for heart failure through item deduction. The criteria for syndrome differentiation were constructed according to the loading coefficients of the relevant items in the corresponding components based on the clinical data and further analyzed with corresponding TCM theories.

#### Comparing derived syndromes between the proposed model and expert consensus

We compared the derived syndromes from the proposed model and the expert consensus [12]. There are three primary syndrome types in the expert consensus: (1) Qi Deficiency with Blood Stasis, (2) Yang Deficiency with Blood Stasis, and (3) Qi-Yin Deficiency with Blood Stasis. All three syndrome types could be accompanied by Phlegm Retention Syndrome. Thus, the primary and associated syndromes could be separated into five syndrome elements, including Qi Deficiency, Yang Deficiency, Yin Deficiency, Phlegm Retention, and Blood Stasis. The details of the criteria for syndrome differentiation of HF from the expert consensus are shown in Additional file 1: Fig. S1.

#### Verification of the derived syndromes with tongue analysis

**Fig. 2:**
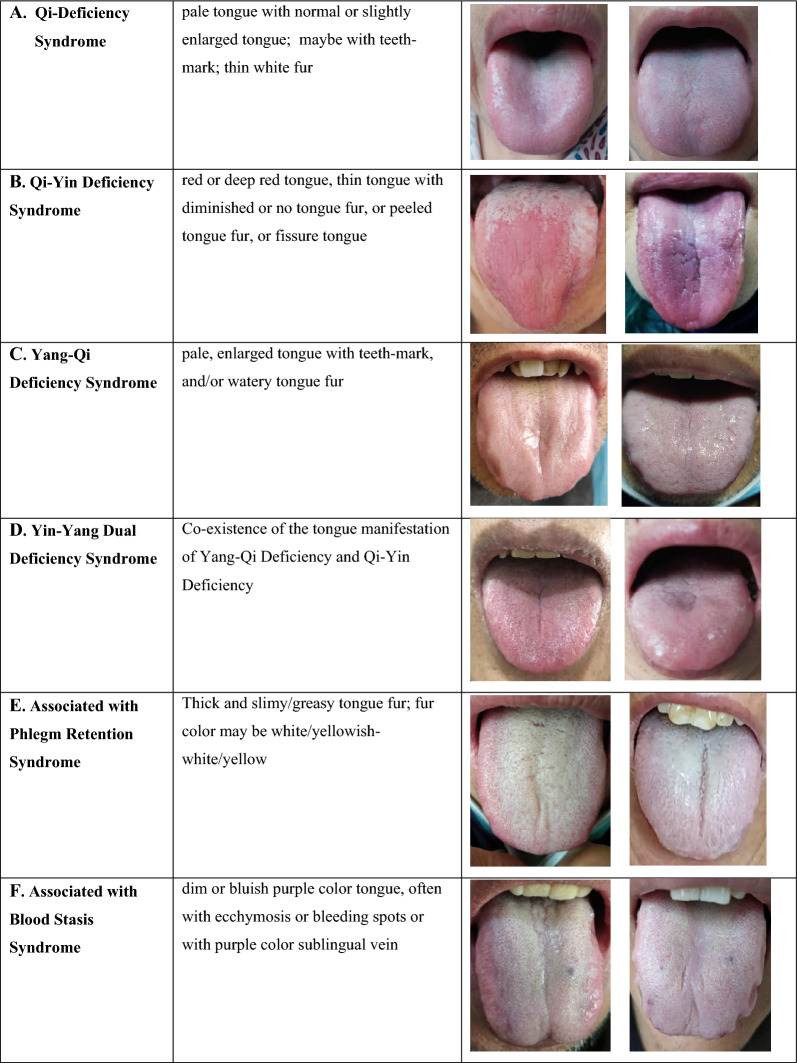
Representative tongue imaging of different syndrome patterns in heart failure patients

Tongue diagnosis is crucial in TCM syndrome differentiation. Representative tongue imaging pictures of different syndrome patterns are shown in Fig. 2. The tongue property of Yang-Qi Deficiency is characterized by the pale tongue color, the enlarged sizes surrounding teeth marks, with watery and slippery white fur. The tongue property of Yin Deficiency shows deep red color inclining to purple, thin and shriveled tongue sizes with detached fur or no tongue fur. The tongue property of Blood Stasis reveals purple or dark purple tongue color, companies with purple spots or bruises, etc. Thus, we acquired the tongue images of these patients and added tongue diagnosis in the SDQHF. The investigators captured the tongue images by using the same brand and model of imaging capturing device. The tongue images were centrally analyzed by an independent team, including 4 TCM practitioners with 3 to 30 years of experience through a well-defined 2-level review process to ensure the accuracy and reliability of tongue analysis. This method minimized the potential subjective interpretation from TCM practitioners due to different experience levels and the possibility of visual discrepancies. The syndromes analyzed from the tongue analysis were related to the derived syndromes of the proposed model and expert consensus, with a particular emphasis on distinguishing empirical statements from rigorous theoretical results [23, 24].

#### Conducting gap analysis on the derived syndromes with tongue analysis

A gap analysis was performed to identify the discrepancy between the derived syndromes of the proposed model and the tongue analysis. The objective was to investigate the underlying reasons for the difference and to mitigate the gap with supportive TCM theory and clinical evidence. Final syndromes were concluded upon the gap analysis.

#### Finalizing the diagnostic criteria for syndrome differentiation and proposing refinement to expert consensus (if any)

The diagnostic criteria for syndrome differentiation of HF for the proposed model were finalized, including the observations from the tongue analysis. Based on the findings, recommendations were made to the expert consensus for efficacy improvement.

### Data management

The patient’s demographic and clinical data were recorded and kept in the CRF folder, then inputted into Epidata software for centralization. Tongue images were sent to a dedicated mailbox and kept in a separate image database. The data management process complied with the regulatory requirements of Clinical Trial Quality Management Regulations and Clinical Trial Data Management Work Technical Guidelines to ensure data authenticity, integrality, accuracy, and traceability.

### Data analysis and statistical methodology

The TCM Questionnaire was analyzed with SPSS 24.0 (Chicago, IL, USA). Data was presented by Mean ± S.D. A descriptive analysis was first performed on the collected data to understand the participants' demographic characteristics and the patients' distribution according to the NYHF functional classifications. The reliability of the SDQHF was evaluated by Cronbach α coefficient and the average inter-item correlation for internal consistency. Factor analysis was applied to examine the content construct by extracting core factors based on identifying the component of the syndrome elements. The ability and accuracy of the SDQHF to derive the syndromes for HF were verified with the TCM syndrome from tongue analysis.

## Results

### Pretest

The panel of subject matter experts provided positive feedback on the pretest. The list of clinical manifestations of heart failure was appropriate for the diagnosis and syndrome differentiation of heart failure. The terminologies used in the questionnaire were easy to understand by general physicians and patients with no TCM background, and the flow of the questions was smooth. No content change was required; only minimal modifications were made to fine-tune several words.

### Feasibility assessment

A simplified version of the 'traffic light' system was adopted to obtain feedback from the patients and interviewers on the feasibility of the SDQHF and the interview flow [25, 26]. The feasibility assessment was performed to evaluate the questionnaire. The gross response rate was 100%. All questionnaires were completed within 10 min. There was only less than 1% of questionnaires that contained missing values. Positive feedback was received from patients and interviewers regarding the simplicity and viability of the questionnaire, the flow of questions, etc.

### Reliability assessment

Internal consistency of the 27 items with a 4-point scale in the SDQHF was examined by Cronbach's α and the average inter-item correlation [27]. Nine items were excluded due to the different scale points or quantitative nature. The exclusive items included the texture and color of phlegm, frequency and amount of urination, face and lip color, location(s) of edema, cold sweating, and distention of the jugular vein. The overall Cronbach's α was 0.863, indicating high reliability. The inter-item correlation was 0.18. Thus, the SDQHF is reliable and has good internal consistency according to the standards [27, 28].

### Validity assessment

The SDQHF was evaluated by a panel of 4 medical experts, including a general physician, a cardiologist, and senior TCM practitioners, and modified to ensure a clear and consistent understanding for both patients and interviewers. We evaluated the content and construct validity according to the previous report [16]. Content validity is a representative theoretical construct in disease diagnosis and syndrome differentiation. The construct validity was assessed to evaluate the variables of the SDQHF and the consistency of syndrome differentiation for heart failure. Factor analysis was conducted using principal component analysis (PCA) and the varimax rotation method to verify the construct validity. The results were satisfactory with the criteria of construct validity, including both the discriminant validity (loading of at least 0.40, no cross-loading of items above 0.40) and convergent validity (eigenvalues of 1, loading of at least 0.40, items that load on posited constructs) [29]. Using the expert consensus, the SDQHF was 86% sensitive for diagnosing heart failure. As stated in the inclusive requirement of this study, all recruited patients must have an established documented diagnosis of heart failure for at least three months, according to the "Chinese Heart Failure Diagnosis and Treatment Guideline," and should receive at least two weeks of standardized treatment. As such, some enrolled patients might not possess the typical clinical manifestations of HF, thus, affecting the sensitivity of the questionnaire. Because all the patients had heart failure, the questionnaire's specificity might not apply to our study.

### Construction of the criteria for syndrome differentiation

**Fig. 3:**
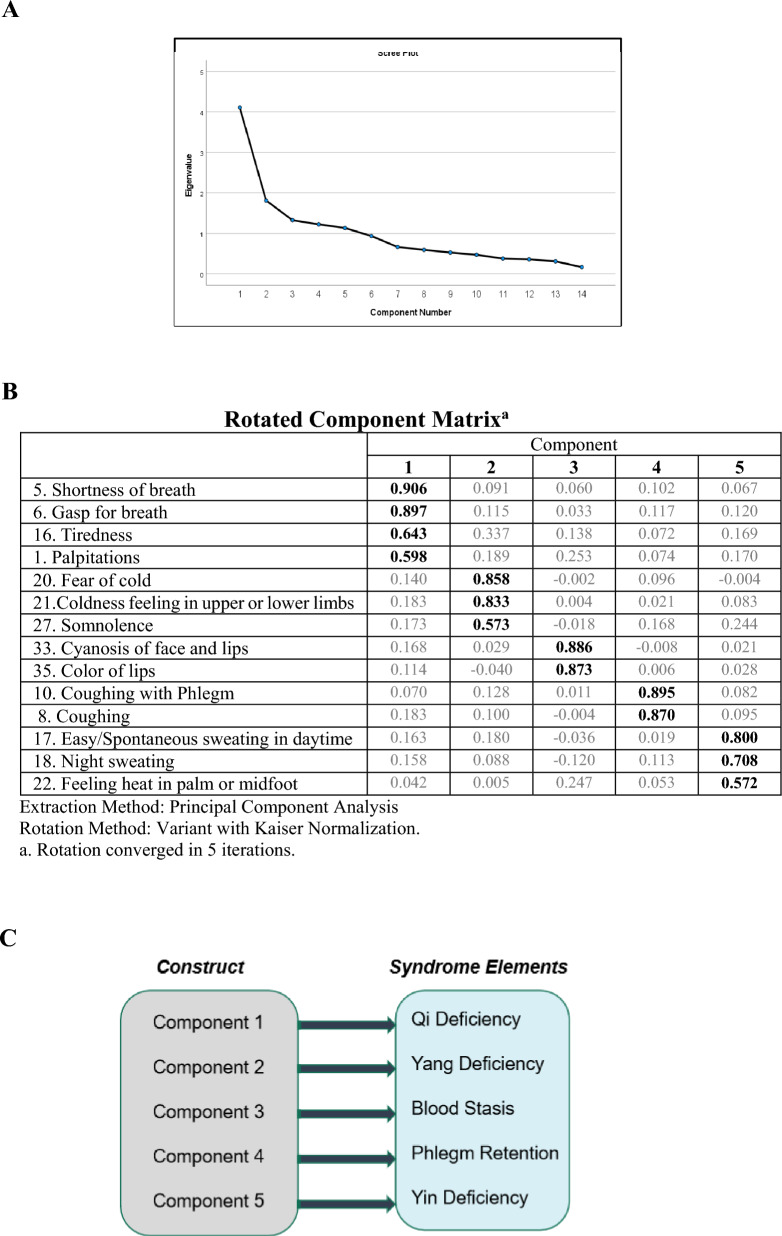
PCA for construction of criteria for syndrome differentiation in heart failure patients. **A** Scree plot diagram of the proposed model: 5 components were identified with eigenvalue ≥ 1. **B** Rotation component matrix of the proposed model and the mapping of the 5 components. **C** Mapping of the 5 components to the underlying theory. With the underlying TCM theory of Collateral Disease of Heart failure, all 5 components could be mapped to the corresponding syndrome element: Component 1 could be mapped to Qi Deficiency; Component 2 could be mapped to Yang Deficiency; Component 3 could be mapped to Blood Stasis; Component 4 could be mapped to Phlegm Retention, and Component 5 could be mapped to Yin Deficiency

PCA was used to construct the significant variables of HF syndromes, reducing variables but maximizing their interpretability. Five-component models with 14 items were identified as the best-fit approach for syndrome differentiation of heart failure. The calculated KMO value of this model was 0.756, and Bartlett's test of Sphericity showed the correlation matrix 3100.513 with p < 0.001, indicating a statistically significant correlation with the PCA criteria. A scree plot was used to predict the number of factors, and five components with an eigenvalue greater than one were extracted (Fig. 3A), which explained 68.6% of the cumulative variance of SDQHF. After Varimax rotation and considering the factor loading with at least 0.4, 14 items forming five components were identified, with item loading ranging from 0.572 to 0.906 (Fig. 3B).

Components from the five-component model indicated an optimal fit for syndrome differentiation of heart failure with the underlying theory of collateral disease. Component 1, corresponding to Qi Deficiency, was measured by three clinical manifestations (palpitations, shortness of breath, and gasp for breath); Component 2, corresponding to Yang Deficiency, was measured by three clinical manifestations (fear of cold, cold feeling in upper or lower limbs, somnolence); Component 3, corresponding to Blood Stasis was measured by two clinical manifestations (cyanosis of face and lips, color of lips); Component 4, corresponding to Phlegm Retention or turbidity was measured by two clinical manifestations (coughing, phlegm in the throat with sound); and Component 5, corresponding to Yin Deficiency was measured by three items (easy sweating in the daytime, sweating at night time, feeling the heat in 5 centers). These five components matched the basic syndrome elements of TCM theory for heart failure (Fig. 3C). Combining the contributing variables of these five syndrome elements formulated the fundamental diagnosis criteria for syndrome differentiation of heart failure, deemed a valid measure of the construct.

### Comparison of the PCA between the proposed model and the expert consensus

**Fig. 4:**
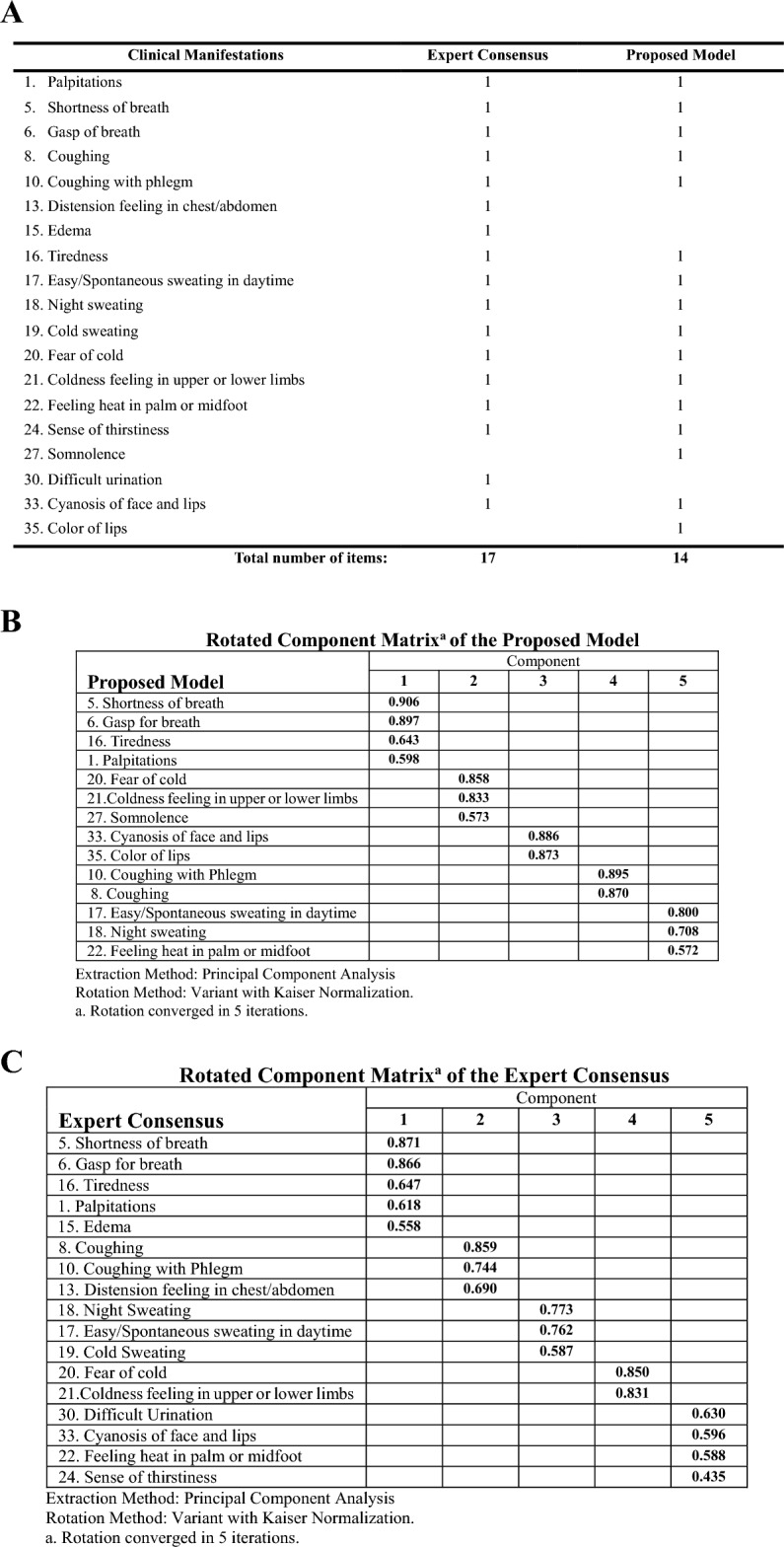
Clinical manifestation items and rotated component matrix for expert consensus and proposed model for syndrome differentiation of heart failure. **A** List of clinical manifestations in the expert consensus and proposed model; **B** Principal Component Analysis of the Proposed model: all 5 components can be mapped to the syndrome elements of HF according to the underlying TCM theories: C1 (Qi Deficiency), C2 (Yang Deficiency), C3 (Blood Stasis); C4 (Phlegm-retention),and C5 (Yin Deficiency) **C** Principal Component Analysis of the expert consensus: Only components 1, 2 & 4 could be mapped to the syndrome elements of HF according to the underlying TCM theories: C1 (Qi Deficiency), C2 (Phlegm retention), C4 (Yang Deficiency)

PCA was conducted with the criteria outlined in the expert consensus for the comparison with the proposed model. The KMO values were 0.780 and 0.755 for the expert consensus and the proposed model, respectively, which fit with the KMO standard. Barlette’s Test of Sphericity was less than 0.05, suggesting a substantial correlation to illustrate the adequate data sets for structure detection. The cumulative variances were 68.574% and 57.626% for the proposed model and the expert consensus, respectively. Thus, the proposed model could represent the variances. With an eigenvalue greater than 1, the expert consensus and the proposed model could extract five components. While all five components of the proposed model mapped to the syndrome elements of heart failure, only two out of five components of the expert consensus could be explained by the underlying TCM theory (Fig. 4).

### Syndrome differentiation from the expert consensus and the proposed model and verification of tongue analysis

As Qi Deficiency is the fundamental cause of heart failure, most patients would have Qi Deficiency syndrome, followed by Yang-Qi Deficiency and Qi-Yin Deficiency. As such, the primary syndrome of HF patients was first derived according to the criteria outlined in the two models. Then, we compared the distributions of the primary syndromes. Hereafter, we cross-verified the derived syndromes from the expert consensus and the proposed model with the results from the tongue analysis to assess their accuracy for syndrome differentiation of HF.

**Fig. 5:**
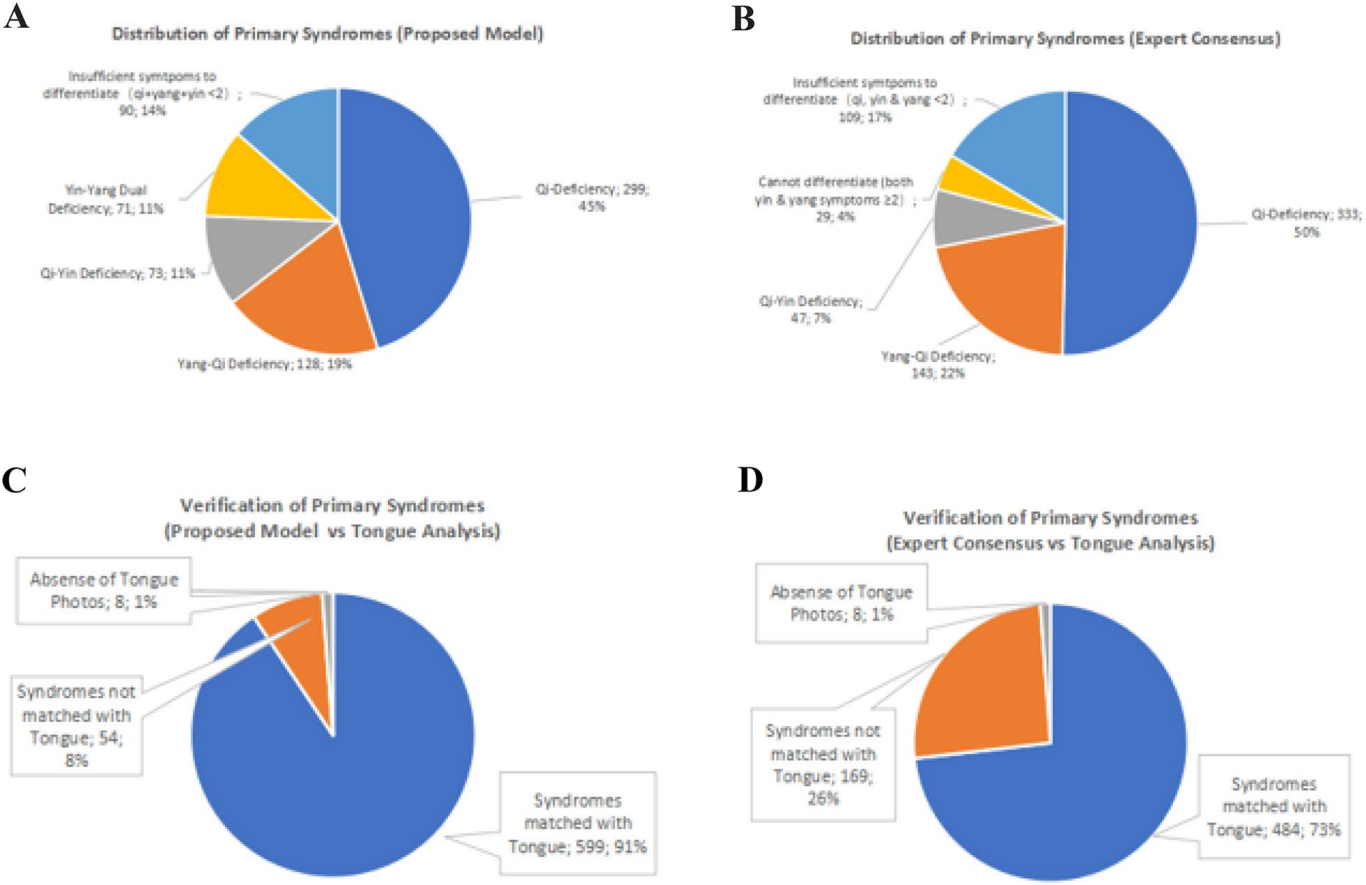
Distribution and verification of primary syndromes derived from proposed model and expert consensus.  **A** Distribution of primary syndromes derived from the proposed model. **B** Distribution of the primary syndromes derived from the expert consensus. **C** Verification of the primary syndromes from the proposed model and tongue analysis. **D** Verification of the primary syndromes from the expert consensus and tongue analysis. Results indicated that compared with the expert consensus, the proposed model has a higher matching percentage of the primary syndrome with the tongue analysis

According to the criteria, the primary syndrome is affirmative, with at least two listed clinical manifestations (syndrome elements). For example, in the proposed model, Qi Deficiency Syndrome was affirmative if the patient had at least two out of four clinical manifestations listed, including shortness of breath, a gasp of breath, palpitation, and tiredness. The same applied to the rest of the primary syndromes. The results showed that the proposed model had similar syndrome distributions to the expert consensus, which matched the TCM theory, but with different contributing percentages (Fig. 5A, B). A substantial portion went to Qi Deficiency Syndrome (45% from the proposed model and 50% from expert consensus), followed by Yang-Qi Deficiency Syndrome (19% from the proposed model and 22% from expert consensus), and finally, Qi-Yin Deficiency Syndromes (11% from proposed model and 7% from expert consensus). In both models, a small portion of the patients with "insufficient symptoms to differentiate" means that the number of clinical manifestations was insufficient to fulfill the syndrome differentiation criteria, thus, needed to be determined by tongue analysis. From the expert consensus, 4% of patients fulfilled the Yin-Yang Dual Deficiency Syndrome. However, there is no such syndrome in the expert consensus; thus, this 4% was left theoretically unclassified.

Neglecting the 1% missing tongue images, the matching percentages between the derived syndromes and the results from the tongue analysis were 91% for the proposed model and 73% for the expert consensus, respectively (Fig. 5C, D). This illustrates that both models are able to derive the primary syndromes for HF, but the proposed model is more competent than the expert consensus in terms of accuracy. Next, a gap analysis revealed 8% discrepancies in the proposed model (Appendix: Table 7). There were 27 samples (21% out of 128) that had mismatched tongue characteristics to the clinical patterns of the Yang-Qi Deficiency Syndrome, showing normal or slightly enlarged tongue sizes, no teeth marks, and no watery tongue fur. Implicitly, this discrepancy in gap analysis could be attributed to the relatively mild clinical manifestations in some patients with Yang-Qi Deficiency Syndrome, leading to untypical tongue features of yang deficiency. The discrepancies for the rest syndromes were comparatively trivial, having a tiny percentage mismatched with the tongue analysis.

**Fig. 6:**
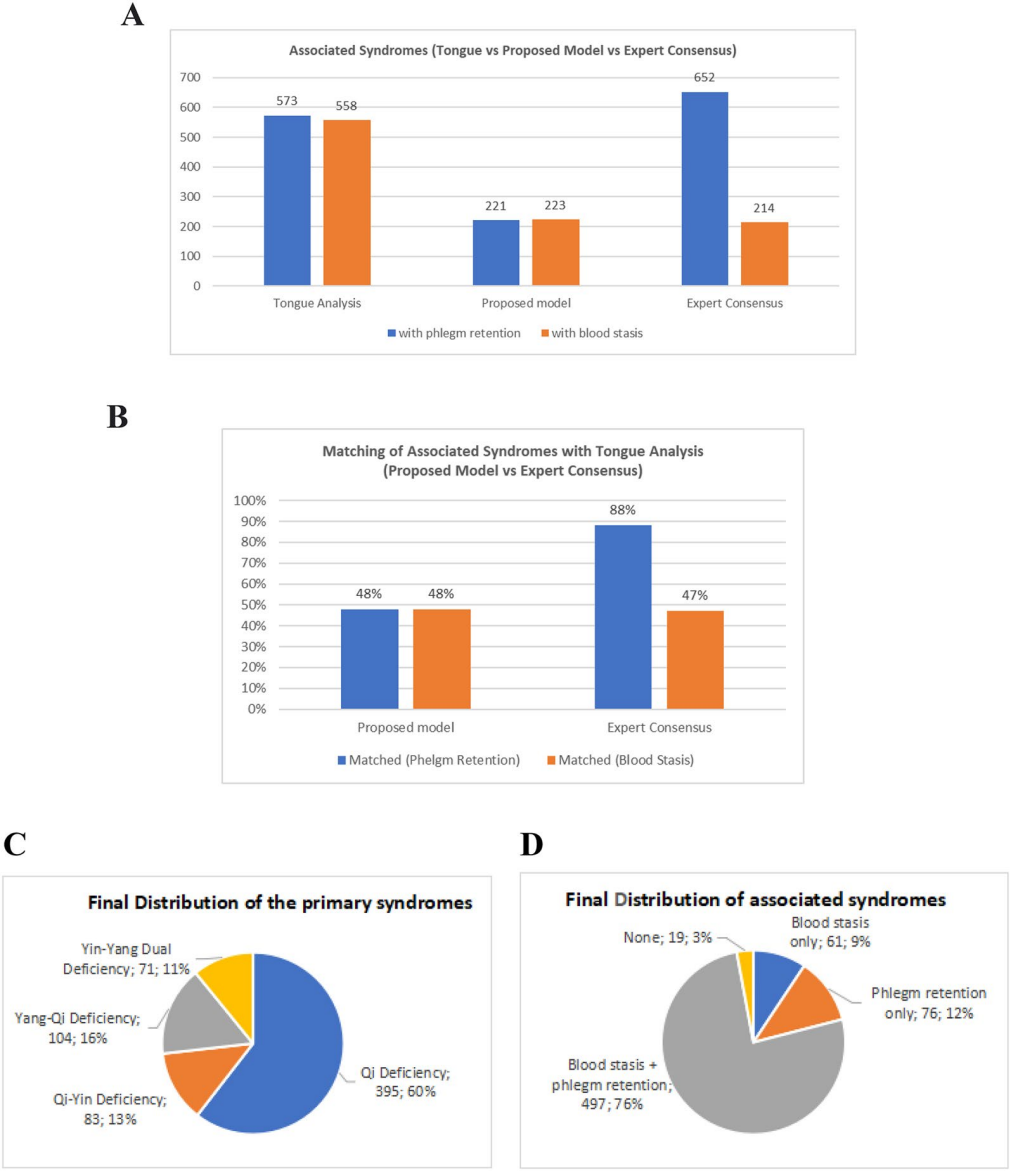
Comparisions of proposed model and expert consensus in associated syndromes and tongue analysis. **A** Distribution of the associated syndromes from tongue analysis, proposed model and expert consensus. **B** Matching of the associated syndromes with the tongue analysis (proposed model vs expert consensus). **C**, **D** Final distribution of the primary and associated syndromes of HF patients

The associated syndromes of HF include Blood Stasis and Phlegm Retention, and the corresponding clinical manifestations of the two models are listed in Table 3. Similar to the primary syndrome, we first derived the associated syndromes from the two models, then compared their distributions, then verified each model's accuracy with results from the tongue analysis (Fig. 6). For Blood Stasis syndrome, 223 (34.2%) patients from the proposed model and 214 (32.8%) from the expert consensus were cataloged with this syndrome, respectively. Notably, the extra variable "color of lip" in the proposed model contributes to an extra nine patients with this syndrome. This suggests that the inclusion of "color of lip" in the proposed model could improve the accuracy of the diagnosis of blood stasis. Theoretically, lips' dim or purplish color is incorporated and highly correlated to "cyanosis of face and lips' for blood stasis syndrome. Practically, it might be easier to observe the blood stasis condition through the inspection of lip color.

**Table 3:** Clinical manifestation variables of proposed model and expert consensus for associated syndromes

Associated syndrome	Proposed model	Expert consensus
Blood stasis	▪ Cyanosis of face and lips ▪ Color of lips	▪ Cyanosis of face and lips
Phlegm retention	▪ Coughing ▪ Coughing with phlegm	▪ Coughing ▪ Coughing with phlegm ▪ Distension feeling in chest/abdomen ▪ Edema ▪ Frequent urination

For the Phlegm Retention Syndrome, two clinical manifestations were listed in the proposed model and five from the expert consensus (Table 3). Coughing and coughing with phlegm are the two common clinical manifestations. Given the significant difference in the number of clinical manifestations between the two models, this ended up with 264 (40.4%) patients from the proposed model and 652 (99.8%) patients from the expert consensus, cataloged with the Phlegm Retention Syndrome, respectively. Indeed, from the tongue analysis, 573 (87.7%) patients fulfilled the tongue manifestations of Phlegm Retention Syndrome. Thus, the gap difference for both models revealed that only relying on clinical manifestations is insufficient to differentiate the Phlegm Retention Syndrome accurately and that tongue manifestation must be included for diagnosis. Notably, from the tongue analysis, the patients with Phlegm Retention Syndrome showed the typical features of watery tongue fur or thin/thick slimy/greasy tongue fur, which is consistent with TCM theories and previous studies [30–32].

### Refinement of syndrome differentiation criteria for the proposed model

**Fig. 7:**
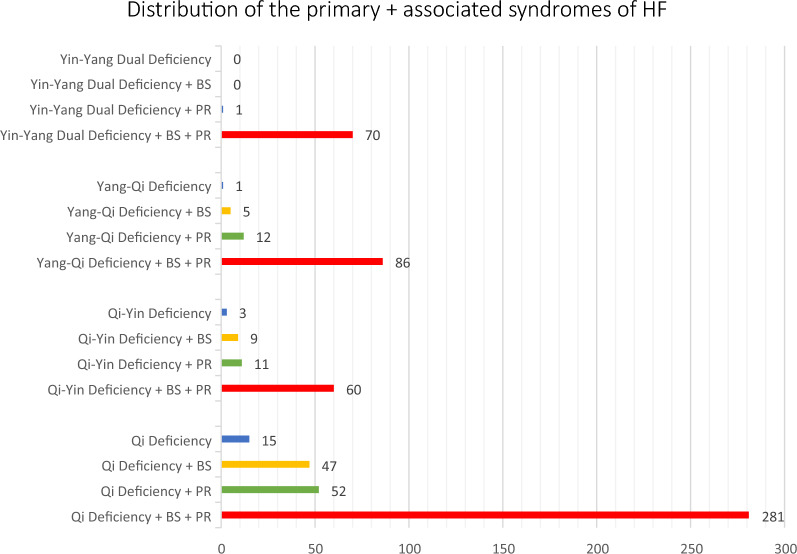
Final distributions of primary and associated syndromes of heart failure patients. BS: Blood Stasis Syndrome; PR: Phlegm Retention Syndrome

Based on the results, we could refine the criteria for syndrome differentiation: (1) The HF patients with less than two clinical manifestations could be cataloged as Qi-Deficiency Syndrome with the confirmation from the tongue features; (2) Yin-Yang Dual Deficiency Syndrome is a justifiable syndrome for HF with supporting clinical evidence, and thus, this syndrome pattern should be included in the criteria as a primary syndrome of HF; (3) Tongue manifestations is crucial for syndrome differentiation of HF, especially for the associated syndrome (i.e., Blood Stasis and Phlegm Retention Retention); (4) If there is a gap between the derived syndrome and tongue analysis, the final syndrome should take the result from tongue analysis. The incorporation of tongue analysis would remarkably increase the accuracy when the data from SDQHF could be prone to survey error, or the symptoms would be suppressed or diminished by medication, and 5) the tongue diagnosis for HF syndrome should be incorporated into the clinical evidence. Based on these findings, the modified diagnostic criteria for the syndrome differentiation of HF are listed in Table 4, and the final distribution of the TCM syndromes of HF is shown in Fig. 7.

**Table 4:** Criteria for HF syndrome differentiation in proposed model

Syndrome		Signs or Symptoms	Diagnosis	Tongue manifestation
Primary syndrome	Qi Deficiency	Palpitations, shortness of breath, gasping for breath, tiredness	At least two signs or symptoms	Normal tongue size, thin-white fur, and/or associated with tender-soft tongue texture
	Yang-Qi Deficiency	Qi Deficiency Syndrome + Yang Deficiency symptoms: fear cold, coldness feeling in upper or lower libs, somnolence	At least two signs or symptoms of yang-deficiency	Enlarged tongue or with teeth-mark, or associated with watery tongue fur
	Qi-Yin Deficiency	Qi Deficiency Syndrome + Yin Deficiency Syndrome: easy/spontaneous sweating during the daytime, night sweating, feeling heat at palm or mid-foot	At least two signs or symptoms of yin deficiency	Red/deep red tongue, thin tongue, diminished or no tongue fur, peeled tongue fur, or fissure tongue
	Yin-Yang Dual Deficiency	Co-existence of the signs and symptoms of Yin Deficiency and Yang Deficiency	At least two signs or symptoms of both Yin and Yang deficiency	Co-existence of the tongue manifestation of Yang-Qi Deficiency or Qi-Yin Deficiency. Example: can be with an enlarged tongue root with thick slimy tongue fur and a relatively thin tongue tip with reduced or no tongue fur
Associated Syndrome	Phlegm Retention	Coughing, Coughing with phlegm	With at least one sign or symptoms+ Tongue manifestation	Watery tongue fur or thin/thick slimy/greasy tongue fur (often thicker in the middle and at the root of the tongue), and may also associate with an enlarged tongue or teeth-marked tongue
	Blood Stasis	Cyanosis of face and lips, dim or purple lip		Dim/purple/bluish purple tongue, or with bruises or bleeding spot

### Recommendations for modified expert consensus

After the analysis and comparison of those two models with 661 HF patients, we would recommend modifying the expert consensus on the diagnostic criteria for syndrome differentiation: (1) To add a new syndrome type, Yin-Yang Dual Deficiency, as the primary syndrome pattern; (2) For Qi-Yin Deficiency Syndrome, the variable "sense of thirstiness" could be removed due to relatively low correlation coefficient to the construct; (3) For Yang-Qi Deficiency Syndrome, the replacement of "cold sweating" with "somnolence" is suggested; (4) For the syndrome differentiation of the associated symptoms like Blood Stasis and Phlegm Retention, the tongue manifestation should be particularly emphasized, and 5) To leverage the tongue manifestation from the proposed model which includes the sophisticated criteria for HF patients. 6) Blood Stasis should be added as an associated syndrome pattern instead of the part of the primary syndrome.

## Discussion

In TCM practice, syndrome differentiation is vital in diagnosing and treating various diseases, including heart failure. Syndrome elements, syndrome types, phenotypic features, and disease form an integral process in the diagnostic path. However, the lacked standardized criteria for syndrome differentiation of heart failure results in a diversified set of syndromes from various clinical studies, leading to difficulties in drawing conclusive outcomes and, thus, hindering the development of clinical studies. To resolve the problems, an expert consensus was proposed in 2014 as a guideline for TCM syndrome differentiation in diagnosing and treating heart failure [12]. Whether the expert consensus can be supported with clinical evidence is a critical scientific question for its use practically. In the present study, we aim to develop an evidence-based questionnaire for the diagnosis of HF based on the expert consensus and construct the criteria for syndrome differentiation of HF. In this multi-center, randomized, double-blind, and placebo-controlled clinical trial, we recruited 661 cases of HF patients to verify the effectiveness of the consensus and the modified questionnaire in SDQHF for HF syndrome differentiation. Our study indicates that the 36-item SDQHF is a reliable and valid instrument for HF diagnosis and syndrome differentiation. Though both the proposed models and the expert consensus are deemed capable of syndrome differentiation of HF, our proposed model is more practical for syndrome differentiation of HF with better matching with the result from the tongue analysis. To our knowledge, this is the first large-scale clinical trial to test the questionnaire and the expert consensus for TCM study. The proposed model for syndrome differentiation for HF and the revised questionnaire can be used for guiding the clinical trials to evaluate the efficacy of TCM treatment in heart failure.

From the distribution of the primary syndromes, the patients with Qi Deficiency Syndrome (60%) are dominant, followed by Yang-Qi Deficiency Syndrome (16%), Qi-Yin Deficiency Syndrome (13%), and Yin-Yang Dual Deficiency Syndrome (11%). Those results suggest that Qi Deficiency Syndrome is the fundamental pathological condition of heart failure patients. The study provides first-hand and robust evidence to support that Qi-Deficiency is the fundamental cause of heart failure in TCM theory. The prolonged Qi Deficiency would be further developed into the status of Yang Deficiency and/or result in different syndrome patterns, including Yang-Qi Deficiency, Qi-Yin Deficiency, and Yin-Yang Dual deficiency [33]. As such, Qi-Yin Deficiency Syndrome and Yang-Qi Deficiency Syndrome should be the progressing stages of heart failure, resulting in the end-stage of Yin-Yang Dual Deficiency Syndrome. Consistently, among the HF patients, the types of Yang-Qi Deficiency Syndrome, Qi-Yin Deficiency Syndrome, and Yin-Yang Dual Deficiency Syndrome were in the proportions of 16%, 13%, and 11%, respectively. To confirm the assumption of TCM syndrome patterns with cardiovascular dysfunctions, we will further explore the correlation between the syndromes and the NYHA functional classification and other biomedical indexes. Though there is a small portion of HF patients with insufficient clinical manifestations to meet the diagnostic criteria for syndrome differentiation, the results from the tongue analysis indicate that all these patients could be classified with Qi Deficiency Syndrome with supportive tongue features. Western prescription medications may suppress the diversity of clinical manifestations of HF. Therefore, flexibility in consideration of clinical manifestations should be considered for the diagnosis of HF.

The associated syndrome of Blood Stasis and Phlegm Retention could be the end-products or the results from the Deficiency of Heart-Qi, Yin, and Yang. Our results showed that 76% of patients had both Blood Stasis Syndrome and Phlegm Retention Syndrome alongside the primary syndromes, 9.4% with only Blood Stasis Syndrome, 11.6% with only Phlegm Retention Syndrome, and 3% of the patients without the associated syndrome. Our study revealed that clinical manifestations should be integrated with tongue diagnosis to enhance the accurate differentiation for Phlegm Retention Syndrome. White slimy/greasy tongue fur, often thicker in the middle and at the root of the tongue, is a prominent tongue feature for Phlegm Retention Syndrome. The formation of the slimy/greasy tongue fur could be due to a particular saliva protein and flow rate, and it is deemed a distinctive characteristic of Phlegm Retention Syndrome for heart disease [31]. As such, tongue diagnosis has merit for accurate syndrome differentiation of HF, especially for the Phlegm-Retention Syndrome.

Furthermore, our study showed that around 15% of HF patients had no symptom and sign related to Blood Stasis Syndrome. The Deficiency of Heart-Qi, Yin, and Yang could lead to the pathological status of Blood Stasis. Thus, Blood Stasis should be an associated syndrome instead of a primary syndrome of HF.

In this study, tongue diagnosis was used to verify the capability and accuracy of the syndrome differentiation. The enrichment and refinement of tongue manifestation facilitate specifically syndrome differentiation in HF patients, which is seldom studied previously. Patients with Yin-Yang Dual Deficiency Syndrome are characterized by an enlarged tongue root with thick slimy tongue fur and a relatively thin tongue tip with reduced or no tongue fur. In addition, the crack(s) existed in patients of various syndrome types, which may be due to the prolonged prescription of diuretics. As such, crack(s) may not be a distinct tongue feature for Yin Deficiency Syndrome in HF patients. We will perform an in-depth tongue analysis to explore the correlation between the special tongue features and the biomedical indexes in the subsequent phases of the study.

Through the validation and analysis of clinical data, we provide several recommendations to modify the criteria of the expert consensus, including [1] To revise various contributing variables for Qi-Yin Deficiency Syndrome and Yang-Qi Deficiency Syndromes: The variable "sense of thirstiness" could be removed from Qi-Yin Deficiency Syndrome; "Somnolence" can be used to replace "cold sweating" for Yang-Qi Deficiency Syndrome; (2) To incorporate the Yin-Yang Dual Deficiency Syndrome as one of the primary syndrome types for heart failure, and also (3) To refine tongue manifestations from tongue analysis. Using PCA, we found that was more clinically related to Qi-Yin Deficiency Syndrome. In general TCM theory, excessive sweating is likely due to Qi or Yang deficiency, which diminishes the control over the sweat pores on sweating. However, for heart failure, this symptom may be more related to the insufficiency of heart blood, which is a form of bodily fluid. Therefore, the symptom of excessive spontaneous sweating during the daytime or exacerbating sweating after activity would be better representative to differentiate the Yin Deficiency Syndrome. Another finding is that somnolence has a higher incidence than cold sweating in heart failure patients with Yang Deficiency Syndrome clinically. Hence, by validating the constructed criteria with clinical data, we could minimize the selection and measurement bias, thus improving the reliability and accuracy of syndrome differentiation for guiding TCM treatment for heart failure patients.

Meanwhile, we note the limitations of this study. Firstly, we have not included pulse diagnosis in the study, although it is a primary method for TCM practitioners to collect clinical information for diagnosis and treatment. The interpretation of pulse diagnosis is often deemed subjective among TCM practitioners, depending on their experiences, finger sensation, and understanding of pulse patterns. It is challenging to obtain consistent recognition and interpretation of pulse patterns by the researchers/interviewers, even for experienced TCM practitioners. Therefore, excluding the pulse diagnosis in the study could eliminate the potential bias on syndrome differentiation of HF unless a reliable and standardized methodology for pulse diagnosis is available, such as the application of Artificial intelligence technology for pulse diagnosis [34]. Without pulse diagnosis information, we should note the potential risks of reducing the reliability of TCM syndrome differentiation. Next, due to the pandemic outbreak of COVID-19 during the assessment period, not all tongue images were captured using the standardized image acquisition device. The patients themselves captured a small portion of tongue images as they could not reach the hospitals during quarantine. However, with our quality control procedures and the well-designed 2-level tongue review process, the impact on the overall quality of tongue analysis would be minimal.

In conclusion, our study demonstrates that the Syndrome Differentiation Questionnaire for Heart Failure (SDQHF) is a valid and reliable method for TCM practitioners to perform syndrome differentiation in diagnosing and treating heart failure. The clinical verification leads us to propose a modified model by changing several symptom items and adding tongue diagnosis as well as Yin Yang Dual Deficiency Syndrome over the expert consensus for the syndrome differentiation of HF. The SDQHF is recommended for further studies in clinical trials to evaluate the efficacy of TCM formulae for heart failure treatment.

### Supplementary Information

**Additional file 1: Figure S1.** Expert Consensus on the criteria for diagnosis and syndrome differentiation of HF [Reference: Guideline for Diagnosis and Treatment of Chronic Heart Failure. Journal of Traditional Chinese Medicine. 2014;55(4):1258-60].

## Data Availability

Not applicable.

## See Tables 5, 6, 7

**Table 5:** Inclusive and exclusive criteria

Inclusion criteria: 1) Provision of signed informed consent prior to any study specific procedures; 2) Male or female, aged ≥ 18 years at the time of consent; 3) Established documented diagnosis of heart failure at least three months ago according to the “Chinese Heart Failure Diagnosis and Treatment Guideline” issued by the Chinese Medical Association Cardiovascular Branch 4) Left ventricular ejection fraction (LVEF) ≤ 40% (echocardiogram, radionuclide, ventriculogram, contrast angiography or cardiac MRI); 5) NYHA cardiac functional grading II to III, with stable clinical symptoms; including those diagnosed as grade IV within 2 weeks before enrollment; 6) Serum NT-proBNP ≥ 450 pg/ml; 7) Those who have received standardized baseline treatment regimens without doses adjusted and given intravenously for at least two weeks prior to enrollment; standardized drug treatment includes: angiotensin-converting enzyme inhibitor (ACEI) or angiotensin receptor blocker (ARB) or angiotensin receptor neprilysin inhibitor (ARNI), beta blocker, and aldosterone receptor antagonist (the optimal therapeutic dose should be achieved, except for contraindications or intolerance);
Exclusion criteria: Patients should not enter the study if any of the following exclusion criteria are fulfilled 1) Heart failure caused by valvular disease, congenital heart disease, pericardial disease, arrhythmia or non-cardiogenic disease, or caused by vital organ failure (such as renal, hepatic failure, etc.); and right heart failure caused by pulmonary or other definite causes; and acute heart failure; 2) Coronary revascularization (percutaneous coronary intervention [PCI] or coronary artery bypass grafting [CABG]) or cardiac synchronization therapy planned to undergo after randomization, or had received cardiac resynchronization therapy prior to enrolment; 3) Any condition outside the CV diseases such as but not limited to malignant tumor, severe mental illness, hematopoietic diseases, neuroendocrine system disease, liver transaminase and alkaline phosphatase ≥ 3 × upper limit of normal (ULN), abnormal renal function serum creatinine > 2 mg/dl (176.82 umol/L), potassium > 5.5 mmol/L; 4) Patient with left ventricular outflow tract obstruction, myocarditis, aortic aneurysm, aortic dissection, or obvious hemodynamic changes caused by unrepaired valve; 5) Cardiogenic shock, uncontrollable malignant arrhythmia, sinus or atrioventricular block at second degree type II or above without pacemaker treatment, progressive unstable angina pectoris or acute myocardial infarction; 6) Uncontrolled hypertension systolic blood pressure (SBP) ≥ 180 mmHg and/or diastolic blood pressure (DBP) ≥ 110 mmHg; or SBP < 90 mmHg and/or DBP < 60 mmHg; 7) Participation in another clinical study with an IP during the last month prior to enrolment; 8) Women of child-bearing potential (i.e., those who are not chemically or surgically sterilized or who are not post-menopausal) who are not willing to use a medically accepted method of contraception that is considered reliable in the judgment of the investigator, from the time of signing the informed consent throughout the study and 4 weeks thereafter, OR women who have a positive pregnancy test at enrolment or randomization OR women who are breastfeeding; 9) Allergic constitution; known to be allergic to research drug; 10) Inability of the patient, in the opinion of the investigator, to understand and/or comply with study medications, procedures or any conditions may render the patient unable to complete the study

**Table 6:** Planned visits and parameters

Visit Schedule	Enrollment & Allocation	Post allocation													Closeout	
	1	2	3	4	5	6	7	8	9	10	11	12	13	14	UNS	EOS
Day/Month	Day0	1 M	3 M	6 M	9 M	12 M	15 M	18 M	21 M	24 M	27 M	30 M	33 M	36 M		
	− 14	± 3	± 7	± 7	± 7	± 7	± 7	± 7	± 7	± 7	± 7	± 7	± 7	± 7		= 2wks
Informed Consent	x															
Inclusion/Exclusion criteria	x															
Randomization	x															
General data and medical history	x															
Medical history of HF	x															
Medical history of CV Diseases	x															
Physical examination	x	x^1^	x^1^	x^1^	x^1^	x	x^1^	x^1^	x^1^	x	x^1^	x^1^	x^1^	x	(x)	x
Heart failure medications	x	x	x	x	x	x	x	x	x	x	x	x	x	x	(x)	x
Medications of other CVD	x	x	x	x	x	x	x	x	x	x	x	x	x	x	(x)	x
Other medications	x	x	x	x	x	x	x	x	x	x	x	x	x	x	(x)	x
TCM Syndrome Diagnosis Questionnaire (SDQHF)	x	x	x			x				x				x		x
Tongue Image Acquisition	x	x	x			x				x				x		x
Echocardiogram	x^*^															
Pregnancy Tests	x					x				x				x	(x)	x
Blood/urine routine test	x	x				x				x				x	(x)	x
Biochemical test	x	x				x				x				x	(x)	x
12-lead ECG	x^*^	x				x				x				x	(x)	x
Serum NT-proBNP at local laboratory	x	x		x												
Dispensing IP	x		x	x	x	x	x	x	x	x	x	x	x	x		
Returning IP for accountability			x	x	x	x	x	x	x	x	x	x	x	x		x
Endpoint Event		x	x	x	x	x	x	x	x	x	x	x	x	x	(x)	x
Adverse Event		x	x	x	x	x	x	x	x	x	x	x	x	x	(x)	x
Serious Adverse Event		x	x	x	x	x	x	x	x	x	x	x	x	x	(x)	x

x^1^Simplified physical examination

x* Cardiac ultrasound and 12-lead ECG within the first 6 months of enrollment

UNS (unplanned visit): (x) The marked item is optional and performed according to the judgment of researchers

EOS (final visit): Make arrangement according to the study end time (if there is a visit within one month before the end of the study, it is regarded as a final visit, but needs to be supplemented with the items required completely)

Pregnancy test is only applicable to women of childbearing age (if the urine pregnancy test is positive, it must be confirmed by serum pregnancy test)

**Table 7:** Gap analysis for primary syndromes of the proposed model with tongue analysis

Primary syndrome derived from the proposed model	No. of samples (n)	Matched with tongue (n)%	Unmatched with tongue (n)%	Unmatched to tongue analysis
*Qi Deficiency (Total symptoms < 2)	90	82 (91)	8 (9)	7 as Qi-Yin Deficiency, 1 as Yang-Qi Deficiency
Qi Deficiency	299	288 (96)	11 (4)	9 as Qi-Yin Deficiency, 2 as Yang-Qi Deficiency
Qi-Yin Deficiency	73	65 (89)	8 (11)	7 as Qi Deficiency, 1 as Yang-Qi Deficiency
Yang-Qi Deficiency	128	101 (79)	27 (21)	24 as Qi Deficiency, 2 as Qi-Yin Deficiency, 1 as Yin-Yang Dual Deficiency
Yin-Yang Dual Deficiency	71	71 (100)	0 (0)	
	661	607 (92)	54 (8)	

*Patients with total symptoms less than 2 in the questionnaire could be classified as Qi Deficiency according to their tongue manifestations

## Abbreviations

HF: Heart failure
NYHA: New York Heart Association
QLQX: Qiliqiangxin capsule
TCM: Traditional Chinese Medicine
SDQHF: Syndrome Diagnosis Questionnaire for heart failure
NT-proBNP: NT-proB-type natriuretic peptide
PCA: Principal component analysis
LVEF: Left ventricular ejection fraction
